# From policy to practice: World Health Organization’s contribution to safer blood transfusions in Africa for sickle cell management – a review

**DOI:** 10.1097/MS9.0000000000004678

**Published:** 2026-01-14

**Authors:** Emmanuel Ifeanyi Obeagu

**Affiliations:** a Department of Biomedical and Laboratory Science, Africa University, Mutare, Zimbabwe; b The Division of Molecular Medicine and Haematology, School of Pathology, Faculty of Health Sciences, University of the Witwatersrand, Johannesburg, South Africa

**Keywords:** Africa, blood transfusion, healthcare services, sickle cell disease, World Health Organization

## Abstract

Sickle cell disease (SCD) is a major public health challenge in Africa, where the prevalence of the condition is high, and it contributes to significant morbidity and mortality, especially among children. Blood transfusions are a crucial therapeutic intervention for managing complications of SCD, such as severe anemia and stroke, which are common among affected individuals. However, many African countries face significant barriers in providing safe, adequate, and timely blood transfusions due to underdeveloped healthcare infrastructure, limited voluntary blood donations, and a shortage of trained healthcare professionals. This review explores the World Health Organization’s (WHO) role in enhancing blood transfusion services for SCD patients in Africa, highlighting its advocacy efforts, policy development, infrastructure strengthening, and capacity-building initiatives. The WHO has been pivotal in advocating for the integration of blood transfusion services into national healthcare systems and developing guidelines for safe blood collection, storage, and distribution. Additionally, WHO has worked to promote voluntary blood donation campaigns, ensuring a steady and safe supply of blood products. WHO’s efforts also include providing technical support to healthcare systems in Africa to improve blood transfusion infrastructure, increase public awareness about the importance of regular blood donation, and ensure that healthcare professionals are properly trained in transfusion medicine. These initiatives aim to enhance the quality and accessibility of blood transfusion services, ultimately improving outcomes for individuals living with SCD.

## Introduction

Sickle cell disease (SCD) is a genetic disorder characterized by the presence of abnormal hemoglobin, which causes red blood cells to adopt a rigid, sickle-like shape. This abnormal shape impairs the cells’ ability to flow smoothly through blood vessels, leading to blockages, pain crises, organ damage, and an increased risk of infections. The burden of SCD is particularly heavy in Sub-Saharan Africa, where an estimated 150 000 children are born with the disease annually. This region also accounts for the highest percentage of global SCD-related mortality and morbidity. Despite the significant impact, the management and treatment of SCD remain suboptimal in many African countries due to a range of healthcare system challenges^[1–4]^. One of the most effective therapeutic strategies for managing the complications of SCD, particularly severe anemia and stroke, is blood transfusion. Blood transfusions provide SCD patients with healthy red blood cells, alleviating anemia, improving oxygen delivery to tissues, and reducing the frequency of painful vaso-occlusive episodes.

**HIGHLIGHTS**

- WHO develops regional policies and guidelines to standardize safe blood transfusion practices.
- Capacity building through WHO-led training programs enhances the skills of healthcare workers in blood screening, storage, and transfusion protocols, ensuring safer services for sickle cell disease (SCD) management.
- WHO supports national blood transfusion services.
- Partnerships and technical support from WHO help African nations implement evidence-based strategies for transfusion safety, directly impacting outcomes in SCD treatment.
- WHO monitors and evaluates transfusion systems, enabling data-driven improvements.

Regular transfusions are especially crucial in managing severe cases and preventing potentially life-threatening complications such as stroke, which disproportionately affects children with SCD. However, providing access to blood transfusions in Africa presents several challenges, including limited access to blood donation services, poor infrastructure, and a lack of trained personnel to safely handle and administer blood products^[5–9]^. The World Health Organization (WHO) has been at the forefront of efforts to improve healthcare delivery across the globe, including strengthening blood transfusion services in Africa. As a key player in global health, the WHO works closely with governments, non-governmental organizations, and international health bodies to ensure the availability, safety, and quality of blood transfusion services. WHO’s involvement in Africa has been critical in addressing the gaps in blood transfusion infrastructure, promoting voluntary blood donation, and supporting countries in their efforts to implement best practices for blood collection, storage, and distribution^[10–12]^.

One of WHO’s significant contributions to the improvement of blood transfusion services in Africa has been its emphasis on strengthening national blood transfusion systems. Many African countries face challenges in establishing robust blood banks, with shortages of blood products being a common issue. WHO has worked to improve blood transfusion systems by providing technical assistance to governments, including the development of blood transfusion policies and the creation of guidelines for blood collection and storage. Additionally, WHO has been instrumental in helping countries strengthen their blood donor recruitment strategies, with a particular focus on increasing voluntary, nonremunerated blood donations, which are considered the safest and most reliable source of blood^[13–15]^. In addition to improving infrastructure, the WHO has made substantial contributions to the safety of blood transfusions in Africa. Blood safety is a major concern in the region due to limited resources for screening donated blood for infectious diseases such as HIV, malaria, and hepatitis. WHO has played a key role in developing guidelines and providing technical support to ensure that blood collection and screening practices meet international safety standards. By implementing these guidelines, WHO aims to reduce the risks associated with transfusions and protect patients from the transmission of bloodborne pathogens, which are a significant concern in many African countries^[16–18]^.

WHO’s capacity-building efforts have also been vital in improving the effectiveness of blood transfusion services. In many African countries, healthcare professionals often lack the necessary training to manage blood transfusions safely. WHO has addressed this gap by providing education and training programs for doctors, nurses, and laboratory technicians involved in blood transfusion practices. These programs aim to ensure that healthcare workers are well-equipped to handle the technical aspects of blood transfusions, manage transfusion reactions, and improve overall patient outcomes. WHO’s capacity-building initiatives are crucial in countries with limited healthcare resources, where staff may not have access to specialized training in transfusion medicine^[19,20]^. In addition to strengthening infrastructure and training healthcare workers, WHO has also been involved in promoting public awareness about the importance of blood donation. Low blood donation rates in many African countries hinder the availability of blood, making it difficult to meet the needs of SCD patients who require regular transfusions. WHO has worked to increase awareness about the critical need for voluntary blood donation, organizing campaigns to encourage citizens to donate blood regularly. These campaigns aim to build a culture of voluntary blood donation, which is essential for ensuring a consistent and safe blood supply for people with SCD^[21,22]^.

## Aim

The aim of this review article is to examine the role of the WHO in enhancing blood transfusion services for SCD patients in Africa.

## WHO’s advocacy and policy development for blood transfusion services in SCD

The WHO plays a pivotal role in advocating for and developing policies to improve blood transfusion services worldwide, with a particular focus on addressing the challenges faced by African nations. Advocacy for blood transfusion services has been integral to WHO’s mission to promote global health and well-being. In many African countries, blood transfusion services are often underdeveloped, with significant gaps in infrastructure, capacity, and safety protocols. The WHO’s advocacy efforts aim to highlight the importance of a robust, accessible, and safe blood supply, especially for vulnerable populations like those living with SCD, who require frequent blood transfusions to manage the disease and its associated complications^[16,23]^. One of the key areas of the WHO’s advocacy is encouraging governments and international organizations to invest in the development of national blood transfusion systems. Many African countries lack comprehensive blood transfusion policies and centralized blood collection, testing, and distribution systems. WHO advocates for the creation of national blood policies that align with international standards to ensure a consistent and safe supply of blood. These policies also emphasize the importance of voluntary, non-remunerated blood donation, as it is the safest and most reliable source of blood. The WHO works to foster political will and government commitment by engaging policymakers and ensuring they understand the long-term benefits of strengthening blood transfusion services, including the reduction of morbidity and mortality from diseases like SCD[24].

WHO’s role in policy development also includes the establishment of technical guidelines and frameworks that shape the implementation of blood transfusion services. The WHO has developed comprehensive guidelines for the collection, screening, storage, and distribution of blood, ensuring that these services meet the highest standards of safety and quality. These guidelines are designed to help countries establish and maintain efficient blood transfusion systems and to ensure that donated blood is free from infectious diseases, such as HIV, malaria, and hepatitis. WHO’s policy development initiatives also emphasize the importance of improving blood transfusion infrastructure, from blood collection centers to storage facilities, and ensuring that blood products are readily available, particularly in regions with a high burden of diseases like SCD. Through these efforts, the WHO is working to create a framework that supports the sustainable provision of safe blood for patients in need[25].

In addition to formal policies, WHO has advocated for increased awareness and education around blood donation and its critical role in healthcare. The WHO’s advocacy campaigns aim to increase voluntary blood donations, which are vital for sustaining blood transfusion services. These campaigns are tailored to address specific cultural and social challenges faced in various African regions, where there may be misconceptions about blood donation or reluctance to donate due to fear of health risks. WHO provides educational resources to help dismantle these barriers, emphasizing the importance of regular blood donation as a communal responsibility that benefits everyone, especially patients with chronic conditions like SCD. By working alongside governments, health organizations, and community leaders, WHO has been instrumental in changing public perceptions about blood donation and encouraging more individuals to donate voluntarily[26].

Furthermore, the WHO plays an essential role in ensuring that blood transfusion policies are integrated into broader national health strategies by aligning blood transfusion services with maternal and child health programs, infectious disease control, and emergency response services. WHO ensures that these services become a key component of a country’s overall health infrastructure. This integration helps to make blood transfusion services more accessible to populations that need them most, including pregnant women at risk of anemia, patients with trauma-related injuries, and individuals suffering from chronic conditions such as SCD. WHO’s advocacy and policy development efforts seek to create a unified approach to healthcare that includes blood transfusion as an integral part of medical care for a variety of conditions[27]. In many African countries, the availability of blood transfusion services is closely tied to the development of healthcare infrastructure. WHO advocates for a systemic approach to healthcare development, which includes strengthening the healthcare workforce, improving medical supply chains, and ensuring that blood transfusion services are well integrated into hospital systems. This holistic approach helps to address the multifaceted challenges faced by countries in ensuring adequate blood supply for SCD patients and other vulnerable groups. WHO’s advocacy for improved healthcare infrastructure also includes facilitating partnerships between governments, nongovernmental organizations, and international health bodies to create sustainable solutions for blood transfusion services (Table 1 and Table 2)[27].

**Table 1:** Summary of WHO’s key policy frameworks and initiatives for blood safety in Africa

Year	Policy/Framework	Core objectives	Key strategies and components	Relevance to sickle cell disease (SCD) management
2000	Global Blood Safety Initiative	Establish safe, adequate, and sustainable blood supplies in all member states	Promotion of voluntary non-remunerated blood donation (VNRBD); universal screening for transfusion-transmissible infections (TTIs); establishment of national blood transfusion services (NBTS)	Provided foundational standards for transfusion safety, essential for chronic transfusion-dependent SCD patients
2002	Global Consultation on Universal Access to Safe Blood	Integrate blood safety into national health systems	Strengthening governance, regulatory oversight, and donor screening practices	Encouraged inclusion of SCD care needs in national blood safety strategies
2008	WHO Regional Committee for Africa Resolution (AFR/RC58/R3)	Strengthen national policies and infrastructures for blood transfusion in the African region	Promotion of centralized NBTS; regional self-sufficiency; establishment of quality management systems	Drove the formation of regional SCD transfusion programs and surveillance networks
2010	Global Collaboration for Blood Safety (GCBS)	Facilitate partnerships for sustainable blood safety programs	Technical cooperation, training, and sharing of best practices among countries	Supported inter-country exchange and standardization of transfusion practices relevant to SCD care
2016	WHO African Blood Safety Network (AfBSN)	Foster regional collaboration and data-driven decision-making	Monitoring, data collection, and capacity building	Strengthened cross-country tracking of blood safety indicators benefiting SCD patients
2020	Action Framework to Advance Universal Access to Safe, Effective, and Quality-Assured Blood Products (2020–2023)	Enhance governance, financing, and innovation in blood services	Strengthening traceability, hemovigilance, and evidence-based donor management systems	Modernized transfusion practices and improved SCD-specific patient safety through better monitoring and evaluation

**Table 2:** Country-level implementation progress in safer transfusions for sickle cell disease

Country	National blood policy status	WHO-supported initiatives implemented	Key achievements/outcomes	Challenges remaining
Ghana	National Blood Policy established (2006) and updated (2020)	WHO-supported quality assurance programs, NBTS centralization, and TTI screening upgrades	Reduced TTI prevalence among SCD patients; improved availability of phenotype-matched blood	Limited rural coverage; dependence on external funding
Nigeria	National Blood Transfusion Service established (2005)	Implementation of WHO VNRBD guidelines; training of transfusion officers; national donor awareness campaigns	Expanded donor pool; improved SCD transfusion protocols in tertiary hospitals	Uneven implementation across states; cold chain gaps
Kenya	National Blood Transfusion Service (est. 2001) with WHO technical input	Digital donor registry; WHO-supported TTI testing kits; hemovigilance reporting	Improved donor retention and traceability; lower infection rates	Inconsistent supply to peripheral hospitals
Rwanda	Early adoption of the WHO’s centralized blood service model	Fully voluntary donation program; WHO quality certification	100% voluntary donations; reliable blood availability for pediatric SCD care	Resource constraints for molecular blood typing
Tanzania	WHO collaboration for strengthening NBTS (since 2012)	Training on blood component preparation; expanded donor recruitment	Improved clinician confidence in transfusion safety for SCD patients	Limited storage capacity; donor retention challenges
South Africa	Longstanding WHO-aligned national service (SANBS)	Regional training hub for blood safety; data sharing with WHO-AfBSN	Benchmark for Africa; provides transfusion safety data supporting WHO programs	High operational costs; sustainability pressures
Uganda	WHO-supported NBTS strengthening (since 2015)	Standardized screening for TTIs; transfusion monitoring in SCD clinics	Improved safety compliance; reduced transfusion errors	Infrastructure and logistics limitations in rural districts

## Strengthening blood collection, storage, and distribution infrastructure

The effective management of blood transfusion services relies heavily on the strength of the infrastructure supporting blood collection, storage, and distribution. In Africa, these essential components of the blood transfusion chain often face significant challenges, including inadequate facilities, insufficient trained personnel, and limited resources. As a result, the WHO has been deeply involved in strengthening blood transfusion infrastructure across the continent, with a focus on enhancing the safety, efficiency, and accessibility of blood products for patients with SCD and other medical conditions that require regular blood transfusions^[28–30]^.

One of the key areas of focus for WHO in strengthening blood collection infrastructure is improving the availability and accessibility of blood donation centers. In many African countries, blood collection is often centralized in urban areas, leaving rural and remote populations underserved. To address this, WHO has advocated for the establishment of mobile blood collection units and regional blood banks that can extend the reach of transfusion services to underserved areas. Mobile blood units are especially crucial in reaching rural communities, where transportation barriers and limited healthcare access often prevent individuals from donating blood. WHO’s support for these mobile units includes providing the necessary equipment, training for staff, and logistical support to ensure that blood donations are collected safely and efficiently^[15,16,31]^.

In addition to improving blood collection, WHO has worked to strengthen blood storage systems across Africa. Proper blood storage is critical to ensuring that blood products remain safe and viable for use in transfusions. Inadequate storage facilities, an unreliable power supply, and a lack of proper refrigeration often result in blood wastage or contamination, which further exacerbates the challenges faced by blood transfusion services in many African nations. WHO has provided technical assistance to help countries build and upgrade blood storage facilities, ensuring that blood can be stored at the appropriate temperatures and that there is a reliable backup power supply. This also involves ensuring that blood is labeled and tracked properly so that it can be distributed efficiently to patients in need^[32,33]^. The distribution of blood products is another critical aspect of the transfusion chain that requires attention. Once blood is collected and stored, it must be transported to hospitals and clinics where it is needed, often under strict timelines and conditions to prevent spoilage. WHO has worked to improve the logistics of blood distribution by helping countries develop supply chain management systems that track blood donations from collection through to transfusion. This includes creating systems for better coordination between blood banks, hospitals, and health centers, as well as implementing technology to monitor blood inventories and ensure timely distribution.

Such systems help to minimize blood shortages and reduce waste by ensuring that blood is used efficiently and effectively^[25,34]^. WHO has also played an important role in ensuring the safety and quality of blood products during the collection, storage, and distribution processes. Blood safety is a critical issue, especially in regions where infectious diseases such as HIV, hepatitis, and malaria are prevalent. WHO has established guidelines for blood screening, testing for infectious agents, and safe storage procedures to minimize the risk of transfusion-transmitted infections. In addition, WHO supports national blood transfusion services (NBTS) in establishing quality control mechanisms, such as regular inspections of blood banks, monitoring of blood storage conditions, and ongoing training for staff. This ensures that blood products are not only collected and stored efficiently but also meet the highest safety standards to protect patients from infections[35].

Another critical aspect of strengthening blood transfusion infrastructure is the establishment of training programs for healthcare workers involved in blood collection, storage, and distribution. Proper training is essential to ensure that blood transfusion services are delivered safely and efficiently. WHO supports countries by providing training programs for blood bank staff, laboratory technicians, and healthcare professionals involved in blood transfusion practices. These training programs cover essential topics such as blood collection techniques, blood typing, cross-matching, blood storage conditions, and transfusion reactions. By enhancing the skills and knowledge of healthcare professionals, WHO ensures that blood transfusion services are conducted according to best practices, minimizing the risks associated with transfusions and improving patient outcomes[36].

WHO’s efforts to strengthen blood transfusion infrastructure also include advocacy for increased investment in blood services by governments and international donors. In many African countries, blood transfusion services remain underfunded, with limited resources allocated to the development of blood banks and transfusion systems. WHO works with governments and international organizations to raise awareness about the importance of investing in blood transfusion services and to secure funding for infrastructure development. This includes supporting the establishment of national blood policies that prioritize funding for blood collection centers, storage facilities, and distribution networks. In some instances, WHO also facilitates public-private partnerships to help fund blood transfusion services and ensure the sustainability of these programs[37].

## Promoting voluntary blood donation and blood safety

Promoting voluntary blood donation is a central aspect of ensuring a steady and safe blood supply for patients in need, including those with SCD in Africa. The WHO has long recognized that a reliable blood supply is essential for saving lives, especially in resource-limited settings. Voluntary, nonremunerated blood donation is the safest way to ensure that blood products are free from transmissible infections and other complications. WHO’s initiatives aim to encourage individuals to donate blood regularly and without financial incentives, making the process sustainable and safe for both the donors and recipients[38]. One of WHO’s key strategies for promoting voluntary blood donation is through global awareness campaigns that emphasize the importance of regular blood donation for health systems. In many African countries, there are significant barriers to voluntary blood donation, including misconceptions about the safety of donating blood, fear of health risks, and cultural stigmas.

WHO works with governments and local organizations to address these barriers by providing educational materials that inform the public about the benefits of blood donation and the safety protocols in place to protect donors and recipients. These campaigns also focus on dispelling myths about blood donation and highlighting its essential role in saving lives, especially for patients suffering from chronic conditions like SCD, trauma, anemia, and other blood-related diseases[39]. WHO also emphasizes the importance of creating a blood donation culture in communities across Africa. By building trust between blood banks, healthcare professionals, and potential blood donors, WHO has facilitated partnerships and collaborations that promote the long-term sustainability of blood transfusion services. One of the major components of this effort is the training of healthcare staff and volunteers who can engage with communities and encourage blood donation. WHO’s guidelines recommend the use of mobile blood donation units to reach rural and remote populations where access to fixed blood donation centers is limited. These mobile units not only help increase the number of donors but also serve as a means to educate people about the need for regular donations and how they can get involved[40].

Ensuring the safety of donated blood is another critical aspect of the WHO’s approach to promoting voluntary blood donation. Blood safety is a global priority, and WHO has developed strict guidelines for the screening, testing, and handling of blood products to reduce the risks of transfusion-transmissible infections (TTIs) such as HIV, hepatitis B and C, malaria, and syphilis. In many African countries, ensuring the safety of blood transfusions is a complex challenge due to limited resources, a lack of proper testing equipment, and the high prevalence of infectious diseases. WHO’s role in strengthening blood safety involves providing technical assistance, capacity-building programs, and access to quality testing and screening technologies. By ensuring that blood is tested for these infections before being distributed, WHO helps reduce the risk of harm to patients receiving blood transfusions, especially those with SCD who require frequent transfusions to manage their condition[41]. WHO’s support in blood safety also includes the development of guidelines and best practices for the handling and storage of blood products. Blood transfusion services must adhere to strict protocols for storing blood at the correct temperatures, maintaining inventories, and tracking blood units from collection to transfusion. Inadequate storage conditions can lead to contamination or spoilage, rendering blood unusable.

WHO provides countries with the tools and guidance needed to establish efficient blood storage and distribution systems that ensure the safety of donated blood. This also includes ensuring that blood products are used optimally and blood wastage is minimized by matching donations with demand and distributing blood in a timely and efficient manner[42]. Another key element of promoting blood safety is the WHO’s work in ensuring the training and certification of blood transfusion staff. Proper training is essential for maintaining the safety of the entire blood donation process, from donor screening and collection to storage and transfusion. WHO helps countries build a strong workforce of trained blood transfusion professionals who understand and implement the best practices for blood safety. This includes providing training on blood donation techniques, screening for potential health risks in donors, blood testing for TTIs, and proper blood storage protocols. By investing in the education and professional development of healthcare workers, WHO ensures that blood donation services are conducted with the highest standards of safety[43].

In addition to these efforts, WHO encourages the establishment of national and regional blood transfusion systems that are well integrated into the broader healthcare infrastructure. WHO’s advocacy and technical support have helped create and strengthen blood transfusion networks in many African countries, ensuring that blood donations are not only collected safely but also distributed efficiently to hospitals and clinics. A well-established blood network reduces the chances of blood shortages, prevents mismanagement, and allows for a more equitable distribution of blood, particularly to those who need it most, such as patients with SCD, pregnant women with complications, and individuals involved in accidents or surgery^[44,45]^.

WHO’s efforts to promote voluntary blood donation and blood safety have also been directed toward improving the quality of blood donation campaigns in Africa. In many cases, the sustainability of blood donation programs depends on continuous public engagement and the building of donor loyalty. WHO has advocated for the introduction of donor recognition programs that acknowledge regular donors and encourage them to return for subsequent donations. By recognizing the contributions of regular blood donors, these programs help foster a culture of selflessness and community engagement, ensuring that blood supply systems are maintained and are able to meet the ongoing needs of patients[46].

## Enhancing training and capacity building in blood transfusion services for sickle cell patients in Africa

The effective management of blood transfusion services, particularly for SCD patients in Africa, depends on a well-trained workforce capable of providing high-quality care. Blood transfusions are a key therapeutic strategy for individuals with SCD, as they help to manage complications such as anemia, vaso-occlusive crises, and stroke. However, despite the crucial role of transfusions in managing SCD, many African countries face significant challenges in the training and capacity building of healthcare professionals involved in blood transfusion services. The WHO has been at the forefront of addressing these challenges by promoting training programs that enhance the skills and knowledge of healthcare workers involved in blood collection, processing, and transfusion^[47,48]^.

A critical component of the WHO’s strategy for enhancing capacity in blood transfusion services is the development and implementation of specialized training programs for healthcare professionals. These training programs are designed to ensure that clinicians, laboratory technicians, blood bank staff, and other medical personnel possess the necessary skills to handle the complexities associated with transfusions for SCD patients. WHO provides countries with access to training curricula, educational materials, and technical assistance to build expertise in the safe and efficient management of blood products. These training programs cover a wide range of topics, including blood donor recruitment, screening for infectious diseases, blood typing and crossmatching, and managing transfusion reactions. By equipping healthcare workers with the knowledge and skills to deliver optimal transfusion care, WHO ensures that patients with SCD receive the best possible treatment^[49,50]^.

In addition to healthcare provider training, WHO also emphasizes the importance of training blood bank staff in the management and processing of blood donations. Blood banks play a critical role in the care of SCD patients, as they are responsible for ensuring that blood products are safely collected, stored, and distributed. Proper blood bank management involves maintaining strict quality control standards, managing inventory to avoid shortages or wastage, and ensuring that blood is stored under appropriate conditions to prevent contamination or spoilage. WHO’s capacity-building efforts in this area focus on providing blood bank staff with the necessary skills to adhere to international standards for blood safety, including screening for transmissible infections, handling and storage protocols, and implementing efficient blood distribution systems. Training blood bank staff also includes educating them on how to tailor blood transfusion services to the specific needs of SCD patients, who often require regular blood exchanges^[51,52]^.

Another key focus of WHO’s capacity-building efforts is fostering leadership in blood transfusion services. Strong leadership is essential for the successful operation of blood transfusion systems, especially in countries with limited resources. WHO works with national health authorities and local stakeholders to build leadership capacity at both the institutional and national levels. This includes training healthcare leaders in areas such as policy development, blood service management, quality assurance, and ethical issues related to blood transfusions. WHO encourages the establishment of national blood transfusion programs and provides technical support to help countries design and implement policies that prioritize blood transfusion services for SCD patients. By strengthening leadership capacity, WHO helps ensure that blood transfusion services are not only well-managed but also integrated into broader health systems, with a focus on sustainability and scalability[53].

Training and capacity-building efforts also extend to the communities where blood is collected. In many African countries, communities face significant challenges in maintaining a steady supply of voluntary blood donors. WHO’s approach to capacity building includes training community health workers and volunteers who can advocate for blood donation, raise awareness about its importance, and recruit potential donors. WHO promotes the use of mobile blood collection units, which can reach underserved rural areas where traditional blood donation centers may not be accessible. Training community health workers in safe blood collection methods and encouraging them to engage local populations in voluntary donation campaigns helps create a sustainable blood supply system that can meet the needs of SCD patients. Additionally, WHO works with local media and grassroots organizations to raise awareness about SCD and the critical role of blood transfusions in managing the disease, fostering a sense of community responsibility and support for blood donation[54].

WHO’s capacity-building initiatives also include providing access to advanced technologies and equipment for blood transfusion services. Many African countries struggle with outdated or insufficient medical equipment, which hampers the ability to perform safe and effective blood transfusions. WHO supports countries by facilitating access to modern blood collection, testing, and storage equipment, as well as implementing regular maintenance programs to ensure the longevity and functionality of this equipment. WHO also provides training on the use of new technologies, such as automated blood typing and cross-matching systems, which enhance the precision and efficiency of transfusion processes. By equipping blood transfusion centers with advanced technologies and training personnel to use them effectively, WHO helps to improve the overall quality of care for SCD patients[55].

To further strengthen the capacity of blood transfusion services in Africa, WHO advocates for the establishment of national blood transfusion networks that link blood collection centers, blood banks, hospitals, and healthcare facilities. These networks are designed to improve coordination and communication between various components of the blood transfusion system, ensuring that blood products are distributed efficiently and equitably. WHO’s support in developing these networks includes providing guidance on logistics, inventory management, and ensuring that blood is transported under safe conditions. This system ensures that hospitals and clinics serving SCD patients have timely access to the blood products they need, reducing the risk of blood shortages and improving patient outcomes^[56,57]^.

WHO recognizes that capacity building is an ongoing process that requires continuous monitoring and evaluation. WHO supports countries in evaluating the effectiveness of their training programs and blood transfusion systems, identifying areas for improvement, and ensuring that high standards are maintained over time. Regular assessments help identify gaps in training, equipment, and infrastructure, allowing for targeted interventions to address emerging challenges. WHO also facilitates the sharing of best practices and lessons learned between countries, fostering collaboration and the exchange of knowledge to continually improve blood transfusion services across the African continent^[50,58]^.

## Challenges to effective blood transfusion for SCD in Africa

The provision of effective blood transfusion services for SCD patients in Africa faces numerous challenges that impact both the availability and quality of care. These challenges are multifaceted, ranging from logistical and infrastructural barriers to socio-economic and cultural issues. Despite the critical role of blood transfusions in managing SCD, especially in preventing or managing complications such as stroke, vaso-occlusive crises, and severe anemia, these challenges prevent many patients from accessing the necessary treatments in a timely and efficient manner^[13,59]^. One of the most significant challenges to effective blood transfusion services in Africa is the shortage of blood donors. In many African countries, blood donation is often not viewed as a regular or voluntary activity. The stigma associated with donating blood, lack of awareness about the importance of regular donations, and limited trust in the healthcare system contribute to low donor turnout. This problem is particularly acute in rural and underserved areas, where access to blood donation centers is limited, and health literacy may be lower.

SCD patients, who often require frequent blood transfusions to manage their condition, are especially vulnerable to these shortages. Without a consistent and reliable blood supply, many patients face delays in receiving critical care, which can lead to worsening health outcomes and even death^[60,61]^. Inadequate infrastructure and resources also pose significant barriers to effective blood transfusion services. Many African countries struggle with outdated or insufficient medical equipment, particularly in blood banks and transfusion centers. Blood collection, screening, and storage facilities may not have the necessary technology to ensure the safety and quality of blood products. For instance, blood banks in some regions lack automated systems for blood typing and cross-matching, which are essential for ensuring compatibility between donors and recipients. Similarly, the absence of proper refrigeration and storage facilities can lead to contamination or spoilage of donated blood, making it unusable. These infrastructure deficiencies hinder the ability to maintain a safe and efficient blood transfusion service, especially for SCD patients who require regular blood exchanges^[62,63]^.

Another challenge to effective blood transfusion for SCD patients is the prevalence of TTIs in many parts of Africa. While blood transfusion services have improved in recent years, the risk of infections such as HIV, hepatitis B and C, and malaria remains a significant concern in certain areas. Limited resources for screening and testing blood for TTIs can result in unsafe transfusions, which could complicate the health of already vulnerable patients. Though WHO has implemented protocols to ensure the safety of blood donations through rigorous screening and testing, in some settings, there are still gaps in the availability of quality control measures and laboratory infrastructure. The lack of adequate testing equipment and trained personnel in some regions increases the risk of transfusion-related infections, which can have serious, long-term consequences for SCD patients[64]. Logistical challenges also play a critical role in the limitations of blood transfusion services in Africa. Blood transportation from donation centers to hospitals and clinics often faces delays due to poor road infrastructure, unreliable transportation systems, and a lack of appropriate facilities to maintain the blood at the correct temperature during transit.

These delays can result in blood supplies being compromised, either through temperature fluctuations or through the time elapsed between collection and use. Such logistical hurdles can also contribute to blood wastage, especially when transfusions are scheduled based on inaccurate supply forecasts or when blood inventories are not well managed. Effective supply chain management is essential to ensure that blood donations are matched to the patients who need them most, but without the infrastructure to support timely distribution, these efforts are often thwarted[65]. The high cost of blood transfusion services presents another significant challenge. The cost of blood collection, screening, storage, and transportation is often prohibitively high, particularly in resource-poor settings. In many African countries, health systems are underfunded, and public health spending on essential services like blood transfusion is insufficient. As a result, many patients may not be able to afford the costs associated with transfusions, and in some cases, families may need to pay for blood donations or treatments out of pocket. For SCD patients, who require frequent transfusions, this can be a serious barrier to consistent care. In addition, healthcare providers may struggle to meet the demand for blood products due to budget constraints, further exacerbating blood shortages and delays in providing care[66].

Cultural and societal factors also contribute to the challenges surrounding blood transfusions in Africa. In some communities, there are misconceptions and fears about blood donation and transfusions, often due to cultural beliefs, religious teachings, or a lack of understanding. For instance, some people may fear that blood transfusions will alter their identity or that donating blood can cause harm to their health. Additionally, some individuals may have concerns about the safety of receiving blood from unknown donors, especially in areas with high rates of infectious diseases. Overcoming these cultural barriers requires effective community education programs, clear communication about the benefits and safety of blood transfusions, and engagement with local leaders to promote the importance of blood donation[67]. Healthcare workforce shortages and lack of training also complicate the delivery of effective blood transfusion services. In many African countries, there are shortages of qualified healthcare professionals, including doctors, nurses, and laboratory technicians. This shortage is particularly pronounced in rural areas, where healthcare workers may have to handle multiple roles and lack specialized training in transfusion medicine.

Without the necessary skills and expertise, healthcare providers may struggle with blood collection, storage, and transfusion protocols, leading to potential errors and risks for patients. WHO has been working to address these challenges by providing training programs and supporting capacity-building efforts, but the demand for skilled professionals far outweighs the current supply[66]. Inadequate data and monitoring systems for blood transfusion services present challenges for policy development and the efficient allocation of resources. Many African countries lack centralized systems for tracking blood donations, transfusion outcomes, and the overall demand for blood products. This lack of data makes it difficult to forecast blood supply needs accurately, leading to mismatches between supply and demand. Furthermore, without robust monitoring systems, it is challenging to assess the safety and quality of blood transfusions, evaluate the effectiveness of interventions, or identify areas for improvement. Strengthening data collection and monitoring systems is essential for improving the management of blood transfusion services and ensuring that resources are allocated where they are needed most[67].

## Translating policy into practice and the impact on SCD management

The translation of WHO blood transfusion policies into tangible clinical outcomes in Africa marks a crucial step in bridging global health policy with patient-centered practice. Traditionally, many policy frameworks in Africa have struggled to move beyond the conceptual phase due to infrastructural, financial, and governance barriers. However, this review highlights emerging evidence that WHO’s strategic interventions have begun to yield measurable progress in the context of SCD management – demonstrating that well-structured global guidance, when adapted to local realities, can transform health systems and patient outcomes[68]. A novel observation of this review lies in the shift from donor dependence to donor safety and sustainability. WHO’s persistent advocacy for voluntary, non-remunerated blood donation has gradually reshaped blood collection systems in several African nations. The creation of NBTS with WHO technical support has not only improved blood availability but also reduced the historical reliance on family replacement donors – an often unsafe and inconsistent practice. This paradigm shift reflects the operationalization of policy principles into life-saving practices, especially for SCD patients who require multiple transfusions throughout their lifespan[69].

Another distinctive insight is the emergence of disease-specific transfusion strategies within the broader WHO framework. Traditionally, blood transfusion systems in Africa were designed to meet emergency needs such as trauma or obstetric hemorrhage. However, the WHO’s policy influence has stimulated the inclusion of chronic conditions like SCD in transfusion planning. This evolution represents a significant turning point: SCD is now being recognized not only as a genetic disorder but also as a public health priority requiring continuous transfusion safety assurance. In countries such as Ghana and Nigeria, the integration of WHO’s blood safety standards into SCD management protocols has led to reduced TTIs and improved patient survival[69]. Importantly, this review provides new insight into the synergy between policy, health system strengthening, and clinical outcomes. WHO’s approach has fostered a “policy-to-practice ecosystem” where capacity building, laboratory quality control, and workforce training directly translate into better bedside care. For example, the standardization of TTI screening and the adoption of WHO-endorsed quality assurance schemes have significantly lowered infection risks for transfused SCD patients. Furthermore, the establishment of national hemovigilance systems – though still developing – signals a transformative move toward proactive monitoring and evidence-based decision-making.

**Figure 1.**
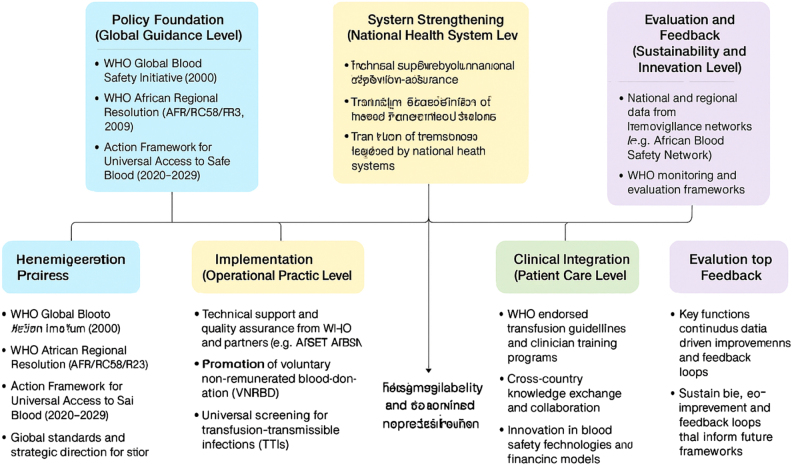
WHO’s policy-to- practice model for safer blood transfusion in sickle cell disease management in Africa.

Another area of novelty is the recognition of regional collaboration as a multiplier of policy effectiveness. WHO-supported networks, such as the African Blood Safety Network, have enabled countries to share best practices, technical expertise, and data on transfusion outcomes in SCD populations. This collaborative framework fosters peer learning and reduces duplication of effort, accelerating policy implementation at both national and regional levels. Such inter-country cooperation is becoming a defining feature of modern public health governance in Africa[70]. This review underscores a contextual innovation – the adaptation of global WHO frameworks to resource-limited African settings. Instead of a one-size-fits-all model, countries are developing context-appropriate solutions aligned with WHO’s core principles. For instance, mobile blood collection drives, use of digital donor registries, and targeted awareness campaigns in schools and faith-based organizations exemplify localized adaptations of WHO’s vision. These community-driven strategies have increased donor retention and strengthened public trust in transfusion systems – factors crucial for sustaining long-term SCD management programs (Fig. 1).

## Novel contributions and practical implications

This review contributes new and valuable insights by moving beyond a descriptive summary of WHO policies to a detailed analysis of how these global frameworks have been *translated into practical, context-specific actions* that directly enhance the safety and effectiveness of blood transfusions for SCD management across Africa. While previous literature has largely focused on the challenges of implementing transfusion safety measures, this work illuminates *how WHO’s strategic interventions have evolved into measurable system-wide improvements* that benefit a vulnerable patient population[67]. A major novel contribution of this review is the identification of a dynamic policy-to-practice continuum – showing how WHO’s blood safety principles have been localized to fit Africa’s health systems and cultural realities. Countries such as Ghana, Nigeria, Kenya, Rwanda, and Tanzania illustrate this transformation through the establishment of functional NBTS guided by WHO standards. These services have introduced coordinated donor recruitment, universal testing for TTIs, and structured distribution systems that now ensure more reliable blood availability for SCD patients. This review, therefore, provides a rare integrative lens linking policy development to field-level health outcomes[68].

Another distinctive element of this review is its focus on disease-specific adaptation. The discussion reveals how WHO’s general transfusion safety frameworks have been reinterpreted to address the unique clinical needs of sickle cell patients, who require recurrent, phenotype-matched, and infection-free blood transfusions. This focus underscores a shift in African health priorities: SCD, once considered a niche genetic concern, is now integrated into national transfusion and public health strategies through WHO’s policy guidance. The review thus highlights WHO’s subtle but profound role in repositioning SCD as a mainstream public health issue[69]. Additionally, this review brings to light context-sensitive innovations that have emerged as by-products of the WHO’s influence. Examples include mobile blood collection campaigns targeting youth populations, community-based donor drives through religious and educational institutions, and the adoption of WHO-endorsed digital donor registries that strengthen traceability and surveillance. These initiatives demonstrate how African nations are creatively adapting global recommendations to overcome infrastructure and cultural barriers – proving that effective policy translation requires flexibility, local ownership, and continuous capacity building[70].

Equally significant is the identification of WHO’s capacity-strengthening legacy in the African blood safety landscape. Through regional training programs, quality assurance workshops, and partnerships with the African Society for Blood Transfusion (AfSBT), WHO has cultivated a cadre of skilled professionals who sustain national transfusion systems. This human resource empowerment represents an often-overlooked but transformative outcome of WHO’s engagement[71]. This review contributes to the academic and policy discourse by proposing a conceptual framework that links WHO’s interventions to measurable outcomes – such as reduced TTIs, improved donor safety, enhanced hemovigilance, and better SCD clinical outcomes. This synthesis bridges the gap between global health policy and clinical hematology, offering a fresh perspective on how organizational leadership can translate into improved patient survival and healthcare resilience.

## Conclusion

The WHO plays a pivotal role in enhancing blood transfusion services for SCD patients in Africa. Through advocacy, policy development, strengthening infrastructure, and promoting safe blood donation, WHO has made substantial contributions toward improving the availability and safety of blood transfusions in the region. The challenges to effective blood transfusion services for SCD patients in Africa are significant but not insurmountable. From blood donor shortages to inadequate infrastructure, logistical issues, and the risk of transfusion-transmitted infections, these obstacles continue to hinder the provision of timely and safe transfusions for those with SCD. However, through concerted efforts by governments, international organizations like the WHO, healthcare providers, and local communities, these barriers can be addressed. Strengthening blood collection, storage, and distribution systems, improving the safety and screening of blood donations, and promoting voluntary blood donation are critical strategies to ensure a reliable blood supply.

Furthermore, improving the capacity of healthcare professionals through targeted training and enhancing the healthcare workforce is essential for the safe and efficient management of blood transfusion services. Equally important is addressing the cultural and socioeconomic factors that may influence blood donation and transfusion practices in African communities. Public awareness campaigns, education, and community engagement are vital to overcoming misconceptions and increasing donor participation. Investments in data collection and monitoring systems will also play a crucial role in improving the efficiency and quality of blood transfusion services by allowing for better resource allocation and timely interventions.

## Data Availability

Not applicable.

## Research registration unique identifying number (UIN)

Not applicable, as this a perspective.

## References

[1] Ojelabi A. Sickle cell disease in Africa. InPublic Health in Sub-Saharan Africa. 2024;2024:140–57.

[2] Ola B Olushola O Ebenso B. Sickle Cell disease and its psychosocial burdens in Africa. InSickle Cell Disease in Sub-Saharan Africa. 2024;2024:67–80.

[3] Obeagu EI Adias TC Obeagu GU. Advancing life: innovative approaches to enhance survival in sickle cell anemia patients. Ann Med Surg (Lond) 2024;86:6021–36. 39359845 10.1097/MS9.0000000000002534 PMC11444627

[4] Obeagu EI Obeagu GU. Management of diabetes mellitus patients with sickle cell anemia: challenges and therapeutic approaches. Medicine (Baltimore) 2024;103:e37941. 38669382 10.1097/MD.0000000000037941 PMC11049766

[5] Obeagu EI Obeagu GU. Living with sickle cell in Uganda: a comprehensive perspective on challenges, coping strategies, and health interventions. Medicine (Baltimore) 2024;103:e41062. 39705436 10.1097/MD.0000000000041062 PMC11666137

[6] Obeagu EI Obeagu GU. Implications of climatic change on sickle cell anemia: a review. Medicine (Baltimore) 2024;103:e37127. 38335412 10.1097/MD.0000000000037127 PMC10860944

[7] Obeagu EI. Strategies for reducing child mortality due to sickle cell disease in uganda: a narrative review. Annals of Medicine and Surgery 2025;87:3279–88. 10.1097/MS9.0000000000002981 PMC12140782 40486550

[8] Ndirangu-Mugo E. Nursing care in sickle cell disease: lessons from East Africa. Sickle Cell Disease in Sub-Saharan Africa. 2024;2024:298–312.

[9] Njoku F Pugh N Brambilla D. Mortality in adults with sickle cell disease: results from the sickle cell disease implementation consortium (SCDIC) registry. Am J Hematol 2024;99:900–09. 38450756 10.1002/ajh.27279 PMC11001513

[10] Obeagu EI. Public–private partnerships in tackling sickle cell disease in Uganda: a narrative review. Ann. Med. Surg. 2025;87:3339–55. 10.1097/MS9.0000000000003082 PMC12140694 40486578

[11] Wonkam A. Perspectives in genomics and sickle cell disease therapeutics. Molecular Hematology. 2024;2024:187–200.

[12] Kiyaga C Ambrose EE Awuonda BO. Building capacity in sub-saharan africa to address sickle cell disease: the consortium on newborn screening in Africa (CONSA). Blood 2024;144:520.

[13] Odame I Bazuaye GN. Transfusions, disease-modifying treatments, and curative therapies for sickle cell anemia in Africa: where are we now? Hematology 2024;2024:234–39. 39643983 10.1182/hematology.2024000550 PMC11665607

[14] Bukini D Rifai A Kanza C. Strengthening advanced therapy for sickle cell disease in Africa: experience from sickle cell disease centre in dar es salaam, tanzania. BMJ Glob Health 2025;10:e017878. 10.1136/bmjgh-2024-017878 PMC11772925 39870485

[15] Jacobs JW Amorim L Pirenne F. The wider perspective: barriers and recommendations for transfusion support for patients with sickle cell disease in low-and middle-income countries. Br J Haematol 2025;206:1585–92. 40147455 10.1111/bjh.20055 PMC12166344

[16] Al-Worafi YM. Sickle cell disease management in developing countries. In: InHandbook of Medical and Health Sciences in Developing Countries: Education, Practice, and Research. Cham: Springer International Publishing; 2024:1–22.

[17] Bell V Varzakas T Psaltopoulou T. Sickle cell disease update: new treatments and challenging nutritional interventions. Nutrients 2024;16:258. 38257151 10.3390/nu16020258 PMC10820494

[18] Munung NS Nnodu OE Moru PO. Looking ahead: ethical and social challenges of somatic gene therapy for sickle cell disease in Africa. Gene Ther 2024;31:202–08. 38012299 10.1038/s41434-023-00429-7 PMC11090833

[19] Zapfel A Thompson A Bridges K. World Coalition on SCD launches, sparking global focus on SCD diagnosis and care. Blood Adv 2023;7:6812–14. 37737740 10.1182/bloodadvances.2023010907 PMC10679800

[20] Hegemann L Narasimhan V Marfo K. Bridging the access gap for comprehensive sickle cell disease management across sub-Saharan Africa: learnings for other global health interventions? Ann Glob Health 2023;89:76. 38025926 10.5334/aogh.4132 PMC10655752

[21] Minja IK Nkya S Bukini D. Strengthening global partnerships for sustainable sickle cell disease care: insights from sickleinAfrica at the 77th united nations general assembly and the US-Africa Leaders. Summit BMJ Global Health 2025;10:e017154. 10.1136/bmjgh-2024-017154 PMC11907018 40086800

[22] Agha RA Mathew G Rashid R. Transparency in the reporting of Artificial Intelligence – the TITAN Guideline. Prem J Sci 2025;10. doi:10.70389/PJS.100082.

[23] Nnodu OE Okeke CO Isa HA. Newborn screening initiatives for sickle cell disease in Africa. Hematology 2024;2024:227–33. 39644044 10.1182/hematology.2024000548 PMC11665715

[24] Paintsil V Ally M Isa H. Development of multi-level standards of care recommendations for sickle cell disease: experience from SickleInAfrica. Front Genet 2023;13:1052179. 36712852 10.3389/fgene.2022.1052179 PMC9877224

[25] Kumar A Bhattacharya S. Sickle cell disease: a comparative perspective on global and national initiatives. Frontiers in Hematology 2024;3:1457158.

[26] Isa H Okocha E Adegoke SA. Strategies to improve healthcare services for patients with sickle cell disease in nigeria: the perspectives of stakeholders. Front Genet 2023;14:1052444. 36816043 10.3389/fgene.2023.1052444 PMC9936139

[27] Smeltzer MP Howell KE Treadwell M. Identifying barriers to evidence-based care for sickle cell disease: results from the sickle cell disease Implementation Consortium cross-sectional survey of healthcare providers in the USA. BMJ Open 2021;11:e050880 10.1136/bmjopen-2021-050880 PMC8601067 34789492

[28] Esoh K Wonkam-Tingang E Wonkam A. Sickle cell disease in sub-Saharan Africa: transferable strategies for prevention and care. Lancet Haematol 2021;8:e744–55. 34481550 10.1016/S2352-3026(21)00191-5

[29] Ally M Balandya E. Current challenges and new approaches to implementing optimal management of sickle cell disease in sub-Saharan Africa. InSeminars in Hematology 2023;60:192–99. 10.1053/j.seminhematol.2023.08.002 PMC10909340 37730472

[30] Dei-Adomakoh Y Asamoah-Akuoko L Appiah B. Safe blood supply in sub-Saharan Africa: challenges and opportunities. Lancet Haematol 2021;8:e770–776. 34481544 10.1016/S2352-3026(21)00209-X

[31] Chirande L Namazzi R Hockenberry M. Building capacity for pediatric hematological diseases in Sub-Saharan Africa. Blood Adv 2025;9:939–47. 39631074 10.1182/bloodadvances.2024012983 PMC11934282

[32] Oreh A. Access to blood products for sickle cell disease warriors in nigeria: challenges and opportunities. Sickle Cell Disease in Sub-Saharan Africa. 2024;2024:313–25.

[33] Nnodu OE Osei-Akoto A Nembaware V. Skills capacity building for health care services and research through the sickle pan African research consortium. Front Genet 2022;13:805806. 35783259 10.3389/fgene.2022.805806 PMC9240392

[34] Inusa BP Atoyebi W Andemariam B. Global burden of transfusion in sickle cell disease. Transfus. Apher. Sci 2023;62:103764. 37541800 10.1016/j.transci.2023.103764

[35] Nsubuga M Mutegeki H Jjingo D. The Ugandan sickle Pan-African research consortium registry: design, development, and lessons. BMC Med Inform Decis Mak 2024;24:212. 39075479 10.1186/s12911-024-02618-9 PMC11285451

[36] Ghafuri DL Greene BC Musa B. Capacity building for primary stroke prevention teams in children living with sickle cell anemia in Africa. Pediatr Neurol 2021;125:9–15. 34563875 10.1016/j.pediatrneurol.2021.08.010 PMC8559257

[37] Dexter D McGann PT. Saving lives through early diagnosis: the promise and role of point of care testing for sickle cell disease. Br J Haematol 2022;196:63–69. 34340260 10.1111/bjh.17678

[38] Anie KA Olayemi E Paintsil V. Sickle cell disease genomics of Africa (SickleGenAfrica) network: ethical framework and initial qualitative findings from community engagement in ghana, nigeria and tanzania. BMJ Open 2021;11:e048208. 10.1136/bmjopen-2020-048208 PMC8311318 34301659

[39] Gammon RR Rosenbaum L Cooke R. Maintaining adequate donations and a sustainable blood supply: lessons learned. Transfusion 2021;61:294–302. 33206404 10.1111/trf.16145 PMC7753343

[40] Kuriri FA. Factors influencing blood donation among young saudi arabian adults: a cross-sectional study to inform donor recruitment and retention programs. Ann. Clin. Lab. Sci 2024;54:224–32. 38802162

[41] Diop S Pirenne F. Transfusion and sickle cell anemia in Africa. Transfus. Clin. Biol 2021;28:143–45. 33515732 10.1016/j.tracli.2021.01.013

[42] Connes P Stauffer E Liem RI. Exercise and training in sickle cell disease: safety, potential benefits, and recommendations. Am J Hematol 2024;99:1988–2001. 39132839 10.1002/ajh.27454

[43] Githiomi R. Distribution OF ABO predicated phenotypes among voluntary blood donors applying next generation sequencing-insights from kenya. J. Med. Biomed. Lab. Sci. Res 2024;4:1.

[44] Benmoussa K Bernaudin F Connes P. Position paper on advancing sickle cell disease management in France by bridging the clinical practices and guidelines through expert insights. Transfusion and Apheresis Science. 2024:103988. 39173314 10.1016/j.transci.2024.103988

[45] Ahmed SG Ibrahim UA. Merits and demerits of sickle cell trait donor blood in tropical transfusion medicine: are there any indications for specific use of blood donated by carriers of sickle cell trait. Afr Sanguine 2021;23:49–59.

[46] Garraud O Politis C Henschler R. Ethics in transfusion medicine: are the intricate layers of ethics all universal? A global view. Transfus. Clin. Biol 2023;30:347–54. 36965847 10.1016/j.tracli.2023.03.004

[47] Ball J Bradley A Le A. Current and future treatments for sickle cell disease-from hematopoietic stem cell transplantation to in vivo gene therapy. Molecular Therapy 2025;33:2172–91. 40083162 10.1016/j.ymthe.2025.03.016 PMC12126839

[48] Musuka HW Iradukunda PG Mano O. Evolving landscape of sickle cell anemia management in Africa: a critical review. Trop. Med. Infect. Dis 2024;9:292. 39728819 10.3390/tropicalmed9120292 PMC11680351

[49] Boma PM Ngoy SK Panda JM. Empowering sickle cell disease care: the rise of technoRehabLab in sub-Saharan Africa for enhanced patient’s perspectives. Frontiers in Rehabilitation Sciences 2024;5:1388855. 38994332 10.3389/fresc.2024.1388855 PMC11236801

[50] Nangunia NM Mukuku O Feza VB. Assessment of healthcare workers’ knowledge and availability of resources for sickle cell disease management in bukavu, democratic republic of the congo. BMC Health Serv Res 2025;25:164. 39875851 10.1186/s12913-025-12330-7 PMC11773787

[51] Dua M Bello-Manga H Carroll YM. Strategies to increase access to basic sickle cell disease care in low-and middle-income countries. Expert Rev Hematol 2022;15:333–44. 35400264 10.1080/17474086.2022.2063116 PMC9442799

[52] Obeagu EI. Potassium dynamics in sickle cell anemia: clinical implications and pathophysiological insights. Ann Med Surg (Lond) 2024;86:6037–45. 39359761 10.1097/MS9.0000000000002551 PMC11444568

[53] Obeagu EI Obeagu GU. Malnutrition in sickle cell anemia: prevalence, impact, and interventions: a Review. Medicine (Baltimore) 2024;103:e38164. 38758879 10.1097/MD.0000000000038164 PMC11098235

[54] Obeagu EI Obeagu GU. Managing gastrointestinal challenges: diarrhea in sickle cell anemia. Medicine (Baltimore) 2024;103:e38075. 38701274 10.1097/MD.0000000000038075 PMC11062666

[55] Obeagu EI Obeagu GU. Telomere dynamics in sickle cell anemia: unraveling molecular aging and disease progression. J Blood Med 2024;15:313–23. 39081620 10.2147/JBM.S462758 PMC11288316

[56] Arji EE Eze UJ Eze Nwaka GO. Evidence-based interventions for reducing sickle cell disease-associated morbidity and mortality in sub-Saharan Africa: a scoping review. SAGE Open Med 2023;11:20503121231197866. 37719166 10.1177/20503121231197866 PMC10504846

[57] Poku BA Pilnick A. Research knowledge transfer to improve the care and support of adolescents with sickle cell disease in Ghana. Health Expectations 2022;25:2515–24. 35909322 10.1111/hex.13573 PMC9615053

[58] Delaney M Telke S Zou S. The BLOODSAFE program: building the future of access to safe blood in Sub-Saharan Africa. Transfusion 2022;62:2282–90. 36173295 10.1111/trf.17091 PMC9643608

[59] Seck M Senghor AB Loum M. Transfusion practice, post-transfusion complications and risk factors in sickle cell disease in senegal, West Africa. Mediterr J Hematol Infect Dis 2022;14:e2022004. 35070211 10.4084/MJHID.2022.004 PMC8746941

[60] Egesa WI Nakalema G Waibi WM. Sickle cell disease in children and adolescents: a review of the historical, clinical, and public health perspective of sub-Saharan Africa and beyond. Int J Pediatr 2022;2022:3885979. 36254264 10.1155/2022/3885979 PMC9569228

[61] Kambale-Kombi P Marini Djang’eing’a R Alworong’a Opara JP. Management of sickle cell disease: current practices and challenges in a northeastern region of the democratic republic of the congo. Hematology 2021;26:199–205. 33594960 10.1080/16078454.2021.1880752

[62] Luzzatto L. Progress and challenges in Africa at the time of molecular haematology. Niger J Haematol 2023;10:e794–e795. 10.1016/S2352-3026(23)00267-3 37793766

[63] Kasai ET Alworong’a Opara JP Ntokamunda Kadima J. Overview of current progress and challenges in diagnosis, and management of pediatric sickle cell disease in democratic republic of the congo. Hematology 2022;27:132–40. 35068390 10.1080/16078454.2021.2023399

[64] Inah GB Ekanem EE Obiora CI. Systemic complications and imaging challenges of sickle cell disease in sub-saharan Africa. J. Radiat. Med. Trop 2021;2:48–54.

[65] Gyamfi J Ojo T Iwelunmor J. Implementation science research for the scale-up of evidence-based interventions for sickle cell disease in Africa: a commentary. Global Health 2021;17:1–4. 33596947 10.1186/s12992-021-00671-x PMC7888072

[66] Soliman AT De Sanctis V Yassin M. Blood transfusion and iron overload in patients with Sickle Cell Disease (SCD): personal experience and a short update of diabetes mellitus occurrence. Acta Bio Medica: Atenei Parmensis 2022;93:e2022291 10.23750/abm.v93i4.13330 PMC9534241 36043959

[67] Mbiya BM Kalombo DK Mukendi YN. Improvement of SCD morbimortality in children: experience in a remote area of an African country. BMC Health Serv Res 2021;21:1–2. 33794895 10.1186/s12913-021-06286-7 PMC8017617

[68] Ojiambo MT. The transatlantic fight against sickle cell disease: an interview with marie ojiambo. Dis Model Mech 2023;16:dmm050629. 10.1242/dmm.050629 PMC10751938 38108235

[69] Bloch EM Vermeulen M Murphy E. Blood transfusion safety in Africa: a literature review of infectious disease and organizational challenges. Transfus Med Rev 2012;26:164–80. 21872426 10.1016/j.tmrv.2011.07.006 PMC3668661

[70] Gondwe A Chipeta E Hosseinipour MC. Facilitators of and barriers to blood donation among voluntary non-remunerated blood donors in sub-Saharan Africa: a scoping review. Vox Sang 2025;120:546–56. 40107721 10.1111/vox.70013 PMC12187535

[71] Loua A Sonoo J Musango L. Blood safety status in WHO African region countries: lessons learnt from mauritius. J Blood Transfus 2017;2017:1970479. 29181226 10.1155/2017/1970479 PMC5664371

